# Efficacy of peroral endoscopic myotomy for the treatment of functional esophagogastric junction outflow obstruction

**DOI:** 10.1016/j.igie.2023.08.003

**Published:** 2023-08-19

**Authors:** Madhusudhan R. Sanaka, Prabhat Kumar, Abdul Mohammed, Rajat Garg, Prashanthi N. Thota, Scott Gabbard, Yi Qin, Monisha Sudarshan, Sudish Murthy, Siva Raja

**Affiliations:** 1 Department of Gastroenterology, Hepatology, and Nutrition, Cleveland Clinic, Cleveland, Ohio, USA; 2 Department of Internal Medicine, Cleveland Clinic, Cleveland, Ohio, USA; 3 Department of Gastroenterology and Hepatology, AdventHealth, Orlando, Florida, USA; 4 Department of Thoracic and Cardiovascular Surgery, Cleveland Clinic, Cleveland, Ohio, USA

## Abstract

**Background and aims:**

Idiopathic or functional esophagogastric junction outflow obstruction (EGJOO) is characterized by preserved peristalsis and an elevated integrated relaxation pressure of the lower esophageal sphincter (LES) without mechanical obstruction. Data on efficacy of peroral endoscopic myotomy (POEM) in EGJOO are limited. Prior studies included heterogeneous patients and those with prior LES interventions such as pneumatic dilation and Heller myotomy. The aim of this study was to determine outcomes of POEM in EGJOO without prior pneumatic dilation or Heller myotomy.

**Methods:**

We reviewed medical records of patients who had POEM for EGJOO between 2014 and 2022 at our institution. The primary outcome was clinical success, defined as a reduction in Eckardt score to ≤3. Secondary outcomes were technical success, adverse events, reduction in LES pressure on high-resolution esophageal manometry (HREM), improvement in timed barium esophagram (TBE), and esophageal pH study findings.

**Results:**

Seventeen patients (9 female subjects; mean age 57.8 ± 14.4 years) met study criteria. POEM was technically successful in all patients, with no significant adverse events. Clinical success was achieved in 94.1% (16 of 17) of patients. There was a significant improvement in barium column height on TBE at 1 and 5 minutes (9.5 ± 6.2 cm vs 2.6 ± 2.6 cm [*P* < .004] and 4.1 ± 5.6 cm vs 1.0 ± 1.6 cm [*P* = .05]) and integrated relaxation pressure of the lower esophageal sphincter on HREM (26.5 ± 9.4 mm Hg vs 6.1 ± 3.6 mm Hg, *P* < .002). The esophageal pH study was abnormal in 5 (41.6%) of 12 patients.

**Conclusions:**

POEM seems to provide high clinical success along with improvement in TBE and HREM in EGJOO; however, GERD is very common after POEM.

Esophagogastric junction outflow obstruction (EGJOO) is a relatively new categorization in esophageal motility disorders with evolving diagnostic criteria. The Chicago Classification Version 4.0 leverages the contractile and pressure patterns seen in high-resolution esophageal manometry (HREM) to classify esophageal motility disorders as disorders of the esophagogastric junction outflow and disorders of peristalsis.^1^ EGJOO is a non-achalasia esophageal motility disorder, characterized by elevated lower esophageal sphincter (LES) median integrated relaxation pressure (IRP) on HREM (Fig. 1) and preserved esophageal peristalsis, confirmed with either an abnormal timed barium esophagogram (TBE) or functional lumen imaging probe.^1^ Clinically relevant EGJOO that may represent an underlying pathologic motor disorder responsive to treatment must have symptoms of dysphagia or non-cardiac chest pain. EGJOO can be secondary to various causes, such as structural, inflammatory, infiltrative, and medication related.^2^ It is considered idiopathic when these causes are ruled out. In mild cases, a trial of conservative management may relieve symptoms.^3^^,^^4^ In patients with more severe or persistent symptoms, targeted LES therapy with botulinum toxin, pneumatic dilation, laparoscopic Heller myotomy, and peroral endoscopic myotomy (POEM) have been described.

**Figure 1:**
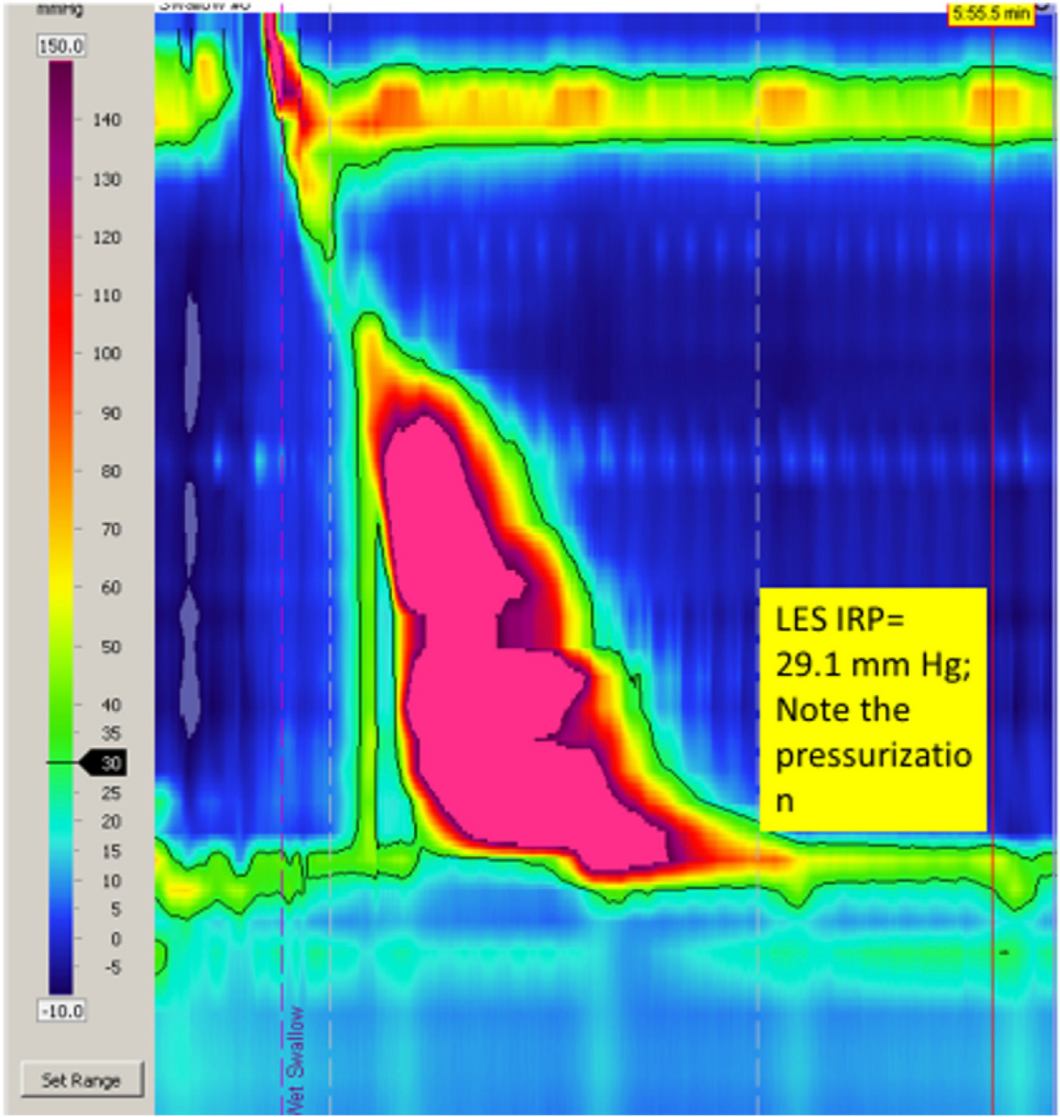
High-resolution esophageal manometry findings in esophagogastric junction outflow obstruction showing lower esophageal sphincter integrated relaxation pressure (LES IRP) of 29.1 mm Hg and preserved esophageal peristalsis.

Recent studies evaluating the efficacy of POEM in the treatment of EGJOO have provided valuable insights.^5^, ^6^, ^7^, ^8^ However, these studies had limitations, including heterogeneous methods for determining clinical outcomes and the inclusion of patients with various motility disorders, as well as those with multiple prior LES interventions. In a systematic review and meta-analysis of 17 studies, Nabi et al^9^ reported a pooled clinical success rate of 77% in the long-term follow-up of patients with non-achalasia esophageal motility disorders, including a subgroup of 17.5% with EGJOO. A prospective study of EGJOO by Ichkhanian et al^10^ reported clinical success in 93% of patients at 6 months’ post-POEM. However, their study had significant limitations. Five (33%) patients had a pneumatic dilation before POEM, and 1 patient had balloon dilation and botulinum toxin injection. Those prior LES interventions might have confounded their study outcomes. Hence, we present the first study to evaluate the efficacy and safety of POEM in managing patients with idiopathic EGJOO without significant prior LES interventions such as pneumatic dilation or Heller myotomy.

## Methods

We reviewed electronic medical records of patients in our POEM registry with EGJOO who had POEM at our institution between April 2014 and October 2022. Our institutional review board approved the study. Consecutive adult patients with symptomatic dysphagia (Eckardt score [ES] >3, measuring the severity of dysphagia, chest pain, regurgitation, and weight loss) with a diagnosis of EGJOO based on esophageal manometry findings according to the Chicago Classification Version 3.0 were included. Before POEM, all patients underwent HREM, TBE, and upper endoscopy. Patients with other esophageal motility disorders, age <18 years, an inability to tolerate general anesthesia, esophagitis, esophageal varices on endoscopy, and coagulopathy were excluded, as were pregnant female subjects. Also excluded were patients with previous significant LES interventions, such as pneumatic dilation and Heller myotomy, before POEM.

### Pre-POEM assessment

**Figure 2:**
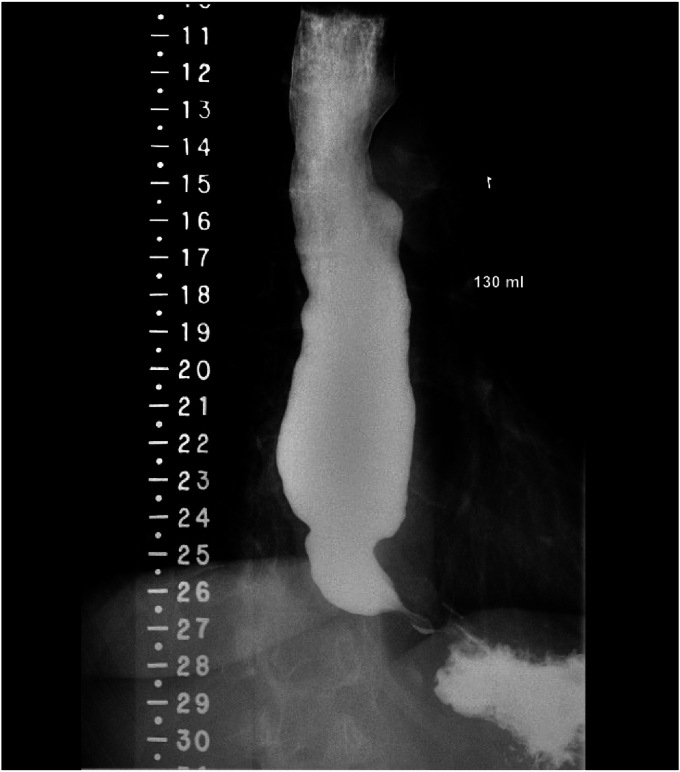
Dilated esophagus with retained barium column in timed barium swallow esophagram.

A POEM baseline ES was recorded before the procedure. HREM at our institution was performed by using the following protocol: a 36-channel, solid-state catheter system with high-fidelity circumferential sensors at 1-cm intervals was advanced through the nasal canal (Sierra Scientific Instruments Inc, Los Angeles, Calif, USA). A computerized analysis system was used to record and analyze pressure data of 10 and 5 mL swallows of water. The Chicago Classification Version 3.0 was used to analyze all relevant parameters. For TBE, patients were instructed to drink the maximum tolerated volume (100-250 mL) of dilute barium sulfate contrast (45% weight in volume) over 30 to 45 seconds in an upright position. Radiographs of the esophagus were taken 1 and 5 minutes after the last swallow, and the height and weight of the barium column were recorded (Fig. 2).

### POEM procedure and postoperative care

**Figure 3:**
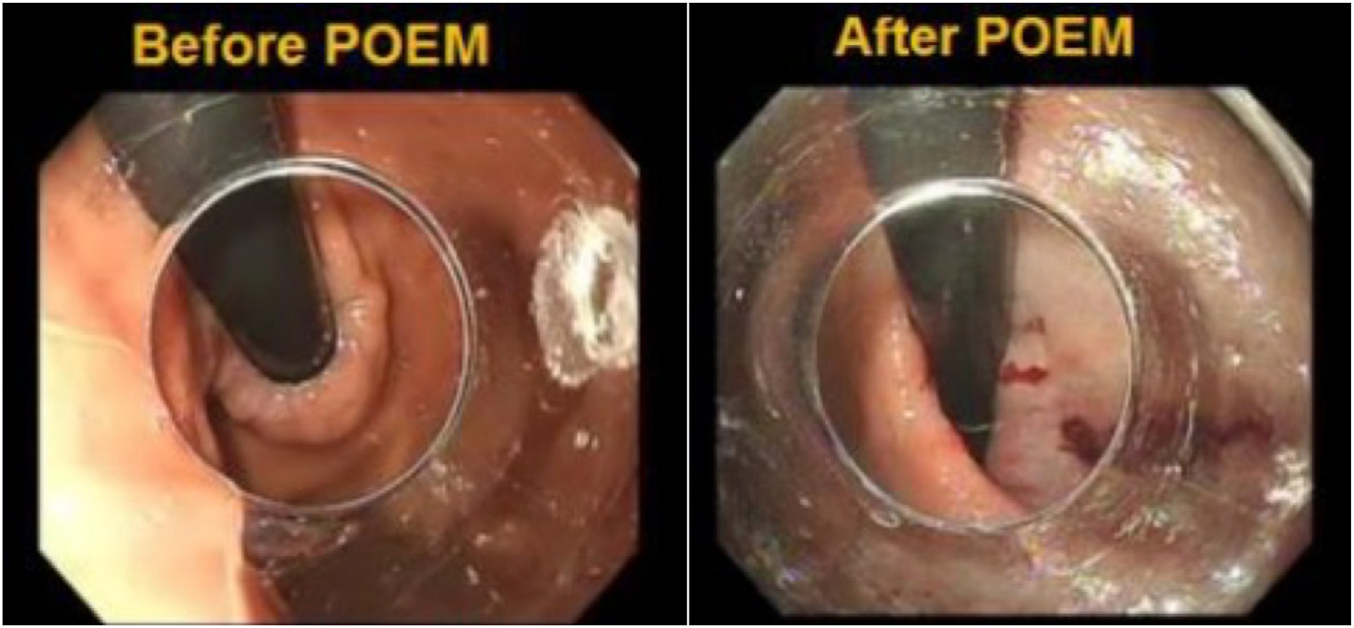
Endoscopic image showing tight gastroesophageal junction before and dilated after peroral endoscopic myotomy (POEM) in esophagogastric junction outflow obstruction.

The patients were nil per os for at least 8 hours before the procedure. POEM was performed with the patient under general anesthesia and with endotracheal intubation. A high-definition gastroscope with a transparent cap mounted on its tip was used for all procedures (Fig. 3). First, an incision was made in the anterior esophageal wall, about 10 cm proximal to the LES, using an endoCUT Q (Erbe, Tübingen, Germany) current with a triangular tip knife (KD 640L, Olympus, Tokyo, Japan). The submucosal space was then accessed, and a submucosal tunnel was created and extended distally along the esophageal wall and extended at least 2 to 3 cm onto the gastric side. A myotomy was then performed, followed by closure of the mucosal incision with endoscopic clips. We typically perform selective circular myotomy in the upper half and full-thickness myotomy in the lower half.

All procedural and postprocedural adverse events were reported and classified according to the American Society for Gastrointestinal Endoscopy (ASGE) lexicon severity scoring system. All patients received periprocedural antibiotics and were observed overnight after the procedure. An esophagogram was obtained the following day to exclude a leak. In the absence of a leak, the patients were started on a clear liquid diet and discharged home with a plan to advance their diet over the next 1 to 3 weeks. Proton pump inhibitor therapy was routinely prescribed after the procedure as a standard practice. If results of the post-POEM pH study were abnormal, proton pump inhibitors were continued long-term.

### Outcomes

The primary outcome measured was clinical success, defined as a decrease in post-POEM ES to ≤3. Secondary outcomes measured were the technical success (defined as successful completion of POEM), reduction in LES pressures on HREM, improvement in TBE parameters at 2 months’ follow-up, and postprocedural adverse events. In addition, GERD post-POEM was recorded using a 24-hour esophageal pH study with a transnasal catheter or Bravo pH study performed 2 months after POEM and off proton pump inhibitors. A total abnormal acid exposure time >5.5% (ie, esophageal pH <4 for >5.5% of the time) was considered as an abnormal pH study.

### Data collection

Preprocedural, procedural, and postprocedural data were abstracted and entered into the POEM Registry in REDCap, maintained by the Cleveland Clinic. All authors had access to the study and approved the final manuscript. Preprocedural data included patient demographic characteristics (age, sex, ethnicity, and body mass index), pre-POEM ES, duration of symptoms, and baseline TBE, HREM, and upper endoscopy findings. Procedural data included procedure date and duration, the extent of myotomy, length of esophageal and gastric myotomies, submucosal tunnel length, and intraprocedural AEs. Postprocedural data included length of hospital stay, postoperative adverse events, treatments needed to manage adverse events, post-POEM ES, TBE, reflux symptoms, esophageal pH study findings, medication (eg, proton pump inhibitors) usage, return to activities of daily living, and length of follow-up.

### Statistical analysis

Appropriate weights were applied for all analyses using STATA version 17 (StataCorp LLC, College Station, Tex, USA). The χ^2^ test and Student t test were used for statistical analysis using SPSS (IBM SPSS Statistics, IBM Corporation, Armonk, NY, USA). *P* values ≤.05 were considered significant. Results are reported as mean ± standard deviation for quantitative variables. Percentages and medians were compared by using the Wilcox rank sum test for categorical variables. Statistical significance was based on 2-sided design-based tests evaluated at α = 0.05. Pre- and post-POEM data were compared.

## Results

### Baseline patient characteristics

Seventeen patients with EGJOO who met the study criteria were included in the final analysis. The mean age of the patients was 57.8 ± 14.4 years. More than one-half (53%) were female (9 of 17), 76.5% (13 of 17) were white, and 23.5% (4 of 17) were African American. The mean body mass index was 31.2 ± 9.3 kg/m^2^. Table 1 presents the baseline characteristics of the study population.

**Table 1:** Baseline characteristics, intraoperative details, and procedural characteristics of POEM in EGJOO

Characteristic	Value
Baseline characteristics and intraoperative details	
Age at POEM, mean ± SD, y	57.8 ± 14.4
Sex, male	8 (47.1%)
White	13 (76.5%)
African American	4 (23.5%)
BMI, mean ± SD, kg/m^2^	31.2 ± 9.3
ASA grade, median (IQR)	3 (1)
Duration of symptoms, mo	88.5 ± 34.64
Procedural characteristics	
Length of procedure, mean ± SD, min	73.4 ± 19.5
Anterior approach	17 (100%)
Myotomy length, mean ± SD, cm	
Esophagus	6 ± 2.2
Stomach	4.1 ± 0.6
Total	10 ± 2.2
No. of clips used, mean ± SD	5.5 ± 1.3
Adverse events	1 (5.8%)

*POEM*, Peroral endoscopic myotomy; *EGJOO*, esophagogastric junction outflow obstruction; *BMI*, body mass index; *SD*, standard deviation; *ASA*, American Society of Anesthesiology; *IQR*, interquartile range.

### Preprocedural findings

Eight (47.1%) of 17 patients were treated with botulinum toxin injection to the LES before POEM. The mean duration of symptoms before POEM was 88.5 ± 34.64 months. All 17 patients underwent preprocedural HREM evaluation for the diagnosis of EGJOO based on the Chicago Classification Version 3.0. The pre-POEM LES mean basal and integrated relaxation pressures were 49.3 ± 21.4 mm Hg and 26.5 ± 9.4 mm Hg, respectively. The pre-POEM mean total ES of 17 patients was 6.4 ± 1.7. Based on TBE findings, the mean esophageal width in 16 of the 17 patients was 1.8 ± .9 cm. Two of the 17 patients had a sigmoidal esophagus. The median American Society of Anesthesiology grade for the study patients was 3 with an interquartile range of 1. Baseline pre-POEM and post-POEM ES, HREM, and TBE findings are presented in Table 2.

**Table 2:** Outcomes of POEM in EGJOO

Eckardt score	Pre-POEM	Median (IQR)	Post-POEM	Median (IQR)	*P* value
	17	7 (3)	16	2 (2)	.001
	Pre-POEM	Mean ± SD	Post-POEM	Mean ± SD	*P* value
	HREM findings (mm Hg)				
LES BMP	17	49.3 ± 21.4	6	17.5 ± 8.4	.004
LES IRP	17	26.5 ± 9.4	6	6.1 ± 3.6	.002
	TBE parameters (cm)				
Height at 1 min	16	9.5 ± 6.2	14	2.6 ± 2.6	.004
Width at 1 min	16	1.8 ± .9	14	1.1 ± 1.3	.13
Height at 5 min	16	4.1 ± 5.6	14	1.0 ± 1.6	.05
Width at 5 min	16	0.8 ± 1.0	14	0.57 ± 1.2	.43

*BMP*, basal mean pressure; *POEM*, Peroral endoscopic myotomy; *EGJOO*, esophagogastric junction outflow obstruction; *IQR*, interquartile range; *LES*, lower esophageal sphincter; *SD*, standard deviation; *HREM*, high-resolution esophageal manometry; *IRP*, integrated relaxation pressure; *TBE*, timed barium esophagogram.

### Procedural details

The POEM procedure was technically successful in all patients (100%), with a mean procedure duration of 73.4 ± 19.5 minutes. The mean esophageal, gastric, and total myotomy lengths were 6 ± 2.2 cm, 4.1 ± 0.6 cm, and 10 ± 2.2 cm, respectively. All 17 patients had a full-thickness anterior approach myotomy. No major intraprocedural adverse events were encountered. One patient had a minor adverse event of mucosal injury on the gastric side, which was closed/approximated using endoscopic clips during the procedure. The length of hospital stay was 1 day, and all patients were discharged the next day.

### Postprocedural findings and outcomes

**Figure 4:**
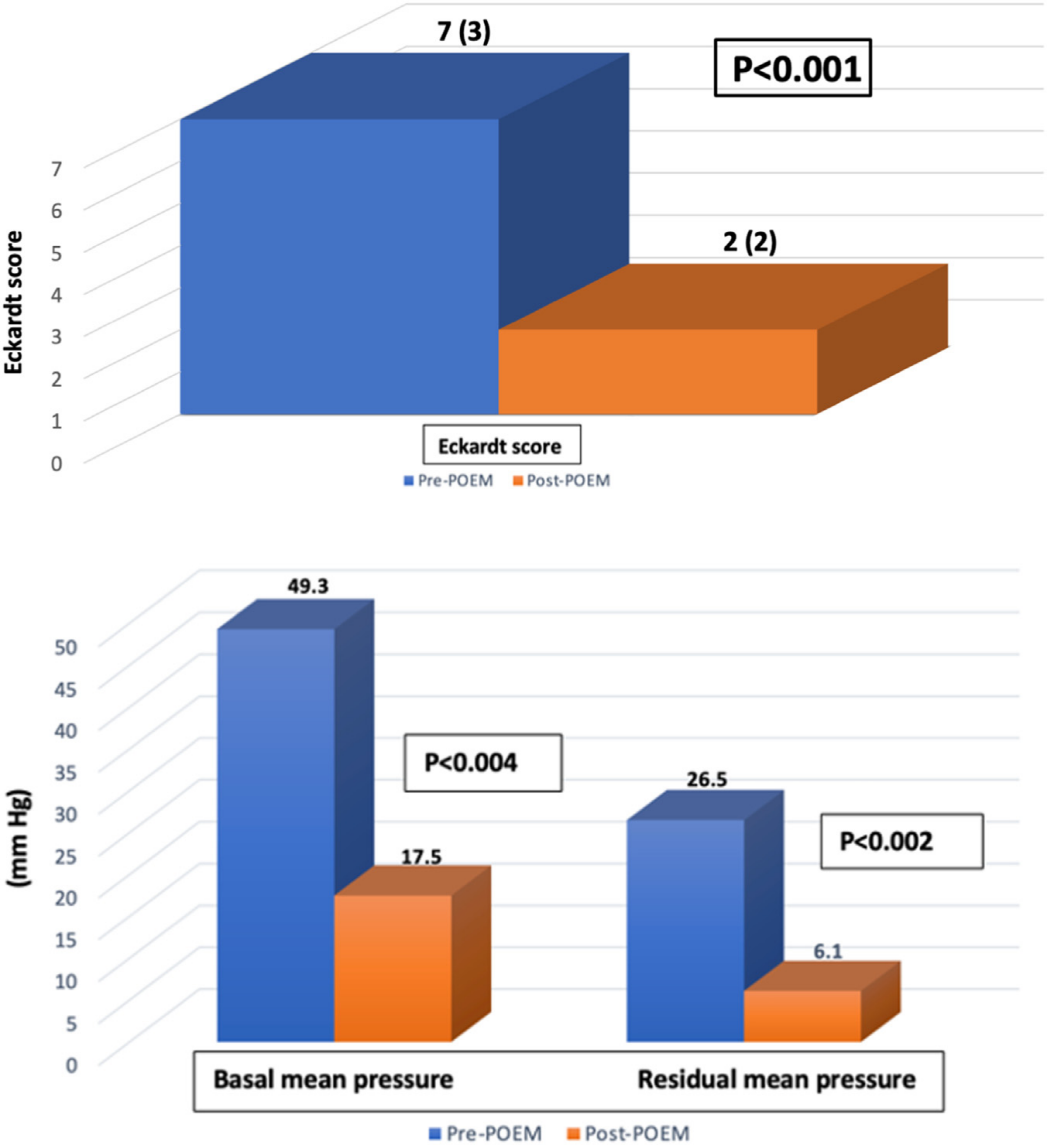
Illustration of Eckardt score, median (interquartile range) and lower esophageal sphincter mean pressures (mean) before and after peroral endoscopic myotomy (POEM).

Clinical success was reported in 94.1% (16 of 17) of the patients, with a significant decrease in the median post-POEM ES (7 [interquartile range, 3] vs 2 [interquartile range, 2], *P* < .001) (Fig. 4). The mean follow-up period to assess clinical success was 78.07 ± 46.57 days. A compilation of the demographic characteristics and outcomes of all 17 patients is shown in Table 3. A total of 6 patients underwent HREM evaluation 2 months after the POEM. Mean integrated relaxation pressure of the LES before and after POEM was 26.5 ± 9.4 mm Hg versus 6.1 ± 3.6 mm Hg (*P* < .002). A total of 14 patients underwent TBE at the 2-month follow-up. There was a significant improvement in the barium column height on TBE at 1 minute (9.5 ± 6.2 cm vs 2.6 ± 2.6 cm, *P* < .004) and at 5 minutes (4.1 ± 5.6 cm vs 1.0 ± 1.6 cm, *P* = .05).

**Table 3:** Patient demographic characteristics and outcomes

Patient	Sex	Age at POEM (y)	BMI (kg/m^2^)	ASA grade	Pre-POEM LES BMP	Pre-POEM LES IRP	Pre-POEM ES	Post-POEM ES	Post-POEM LES BMP	Post-POEM LES IRP	Total % time spent in reflux	Post-POEM EGD findings
1	M	70.35	34	3	58.7	23.7	3	3	6.6	0.5	32.1	Normal
2	M	70.63	24	4	22.4	29.7	4	4	…	…	…	…
3	F	55.32	28	2	37.7	16.8	8	2	21.5	4.6	…	Normal
4	F	52.54	47	2	48.5	28	7	5	8.7	4.5	11.8	LA grade B esophagitis
5	F	50.02	38	3	79.9	40.2	6	1	17.5	6.7	2	…
6	F	47.53	47	3	53.7	28.6	8	0	28.5	9.6	3.7	…
7	F	70.92	23	4	60	22.2	7	1	…	…	16.7	…
8	M	22.84	31	2	64.6	41.3	7	2	…	…	5.3	LA grade A esophagitis
9	M	72.78	20	4	44.8	19.9	5	0	22.2	10.2	0.2	…
10	M	80.66	25	2	42.8	16.4	9	2	…	…	…	Normal
11	F	38.95	38	2	84.1	47.6	7	1	…	…	10.5	Normal
12	M	66.38	25	3	67.2	28.3	6	3	…	…	0	LA grade A esophagitis
13	F	61.1	21	2	21.8	29	6	…	…	…	…	…
14	M	54.4	27	3	22.5	14.2	4	1	…	…	…	…
15	F	61.4	48	2	78.0	26.5	5	2	…	…	4.5	LA grade A esophagitis
16	F	44.9	25.79	3	20.6	16.5	8	2	…	…	12.2	Normal
17	M	61.6	30.23	3	30	22	9	0	…	…	4.7	Normal

*POEM*, Peroral endoscopic myotomy; *BMI*, body mass index; *ASA*, American Society of Anesthesiology; *LES*, lower esophageal sphincter; *BMP*, basal mean pressure; *IRP*, integrated relaxation pressure; *ES*, Eckardt score; *M*, male; *F*, female; *LA*, Los Angeles.

Twelve patients underwent a 24-hour pH study 2 months after POEM. Based on the total abnormal acid exposure time >5.5% (ie, esophageal pH <4 for >5.5% of the time), the post-POEM 24-hour esophageal pH study was abnormal in 41.6% of the patients (5 of 12). A total of 10 patients underwent post-POEM endoscopic evaluation, revealing reflux esophagitis in 4 patients (40%). Three patients had Los Angeles grade A esophagitis, and 1 patient had Los Angeles grade B esophagitis. No other adverse events were observed in any patient.

## Discussion

In this single-center study, we report 94.1% clinical success and 100% technical success rates of POEM in the treatment of idiopathic EGJOO. There was also an improvement in objective measures at follow-up. On HREM, the mean total basal mean pressure at follow-up was 17.5 mm Hg, with a mean decrease in basal mean pressure of 64.5% (31.8 mm Hg), and the mean IRP at follow-up was 6.1 mm Hg, with a mean reduction in IRP of 76.9% (20.4 mm Hg). Also, post-POEM TBE exhibited a significant improvement in barium column height at 1 and 5 minutes. Five (41.6%) of 12 patients had an abnormal esophageal pH study after POEM, whereas 40% of patients who underwent endoscopic evaluation (4 of 10) had Los Angeles grade A or B reflux esophagitis.

POEM is a relatively new approach to targeted LES therapy and is a less invasive alternative to traditional laparoscopic Heller myotomy.^11^ The short- and intermediate-term effectiveness of POEM for treating achalasia has been well documented. In a review of 841 patients who underwent POEM, 82% to 100% of the patients had a posttreatment ES ≤3 and a decrease in integrated relaxation pressure of the LES >50%.^12^ Many studies have also reported >90% improvement in TBE parameters after POEM.^13^, ^14^, ^15^ In several small retrospective studies, POEM has similarly been reported with significant short- and medium-term success in non-achalasia motility disorders.^5^, ^6^, ^7^, ^8^ After POEM, 71% to 100% of patients with EGJOO reported symptomatic improvement in ES. In the study by Filicori et al,^5^ 80% of patients had a sustained clinical response at a median of 48 months after POEM. A prospective study of 14 patients with EGJOO after POEM showed a significant improvement in median total ES at 2 months (6 vs 0, *P* = .02) and 6 months (*P* = .02).^10^ However, only 9 of 14 patients underwent HREM evaluation at a median of 2 months after POEM, and none of the patients underwent TBE. There was a significant decrease in both BRP and IRP with a mean decrease in pressure of 41 mm Hg (*P* = .03) and 17 mm Hg (*P* = .02), respectively. Similarly, our study reports clinical success in 94.1% of the patients with a significant improvement in median total ES (6.4 ± 1.7 vs 1.8 ± 1.4, *P* < .001) at a short-term follow-up duration of 2 to 6 months. Six patients (35.2%) underwent post-POEM HREM, with significant reduction in both BRP and IRP with a mean decrease in pressure of 31.8 (*P* = .004) and 20.4 (*P* = .002), respectively. In our study, there was also significant improvement in barium column heights on TBE after POEM.

POEM is associated with a low incidence of perioperative adverse events when performed by experienced operators.^16^ The potential procedure-related adverse events are aspiration pneumonia, esophageal leak, and pneumothorax; these events are uncommon, however.^17^ GERD and concomitant esophagitis are the more common AEs after POEM. An international multicenter study of a heterogeneous group of 282 patients with achalasia and spastic esophageal disorders reported 25% reflux esophagitis on EGD and 58% abnormal esophageal pH testing on 10- to 24-month follow-up after POEM.^18^ In patients with idiopathic EGJOO, GERD was reported in 30% to 40% of patients after POEM.^6^^,^^19^ In our study, the post-POEM esophageal pH study was similarly abnormal in 41.6% of the patients. Ten patients underwent endoscopic evaluation after POEM; among them, 4 patients (40%) were found to have Los Angeles grade A or B reflux esophagitis.

The current study has a few limitations. First, because EGJOO is not a commonly diagnosed condition, the study included a small number of patients and thus was prone to type II errors. Second, we did not assess the long-term effectiveness of POEM in treating EGJOO. Third, POEM is only available at tertiary care centers with extensive expertise, and thus the results may not be generalizable. Finally, this was a retrospective study. Thus, not all patients completed post-POEM follow-up with HREM, TBE, esophageal pH monitoring, and endoscopic evaluation. HREM aids in understanding the underlying pathophysiology, differentiating EGJOO from other motility disorders. Considering the importance of HREM, we recognize the need for future studies to incorporate a more extensive utilization of this diagnostic modality. However, our study has significant strengths. First, all patients included in the study were clinically symptomatic and underwent objective assessment with HREM and TBE before the procedure. Second, we excluded all patients previously treated with POEM, laparoscopic Heller myotomy, or pneumatic dilation. Thus, we could assess the effectiveness of POEM in managing idiopathic EGJOO exclusively. Finally, despite the registry’s inception in 2014, the patients included in our study meet the updated defining criteria of EGJOO in the Chicago Classification Version 4.0.^1^

In conclusion, POEM seems to be an effective LES-targeted therapy for managing idiopathic EGJOO. Although GERD is a common adverse event after the procedure, it is not prohibitory given the effectiveness of antisecretory medications. Our study can serve as a model for larger, prospective, multicenter trials to assess the short- and long-term effectiveness and safety of POEM in EGJOO. In addition, future studies comparing laparoscopic Heller myotomy with POEM can help determine the preferred treatment strategy in symptomatic patients with EGJOO who have failed conservative management and botulinum toxin injection to the LES.

## Disclosure

All authors disclosed no financial relationships.
